# The Impact of Improving Dermal Permeation on the Efficacy and Targeting of Liposome Nanoparticles as a Potential Treatment for Breast Cancer

**DOI:** 10.3390/pharmaceutics13101633

**Published:** 2021-10-06

**Authors:** Heba F. Salem, Amr Gamal, Haitham Saeed, Alaa S. Tulbah

**Affiliations:** 1 Department of Pharmaceutics and Industrial Pharmacy, Faculty of Pharmacy, Beni-Suef University, Beni-Suef 625617, Egypt; heba_salem111@yahoo.com (H.F.S.); Amr_g@pharm.bsu.edu.eg (A.G.); 2 Clinical Pharmacy Department, Faculty of Pharmacy, Beni-Suef University, Beni-Suef 625617, Egypt; haitham.sedawy@pharm.bsu.edu.eg; 3 Department of Pharmaceutics, College of Pharmacy, Umm Al Qura University, Makkah 21421, Saudi Arabia

**Keywords:** breast cancer, raloxifene, deformable liposomes, propylene glycol, bioavailability

## Abstract

Breast cancer is the most frequent malignancy in women. This work focuses on developing deformable liposomes as a potential carrier for breast cancer treatment and studying the impact of improving dermal permeation on the efficacy and targeting of liposomes. Raloxifene (RXF), an oestrogen antagonist, was used as a model drug. Using Box–Behnken design, different formulations of RXF-loaded deformable liposome (RLDL) were prepared using different propylene glycol, phospholipid and cholesterol concentrations. The percentage of entrapment efficiency (Y_1_), particle size (Y_2_), zeta potential (Y_3_) and steady-state flux (Y_4_) of the prepared formulations were all evaluated. Y_1_ and Y_4_ were significantly increased and Y_2_ and Y_3_ were significantly decreased when the propylene glycol concentration was increased. The optimization was obtained and the optimum formulation was that including phospholipid (1.40% *w*/*w*), cholesterol (0.15% *w*/*w*) and propylene glycol (10% *v*/*v*). The selected optimum formulation displayed a % EE of 78.34 ± 1.04% with a steady-state flux of 4.21 ± 0.02 µg/cm^2^/h. In order to investigate bioavailability, antitumor effectiveness and permeation, the optimum formulation was selected and included in a carbopol gel. The optimum gel formulation had 2.77 times higher bioavailability and, as a result, considerable antitumor action as compared to oral RXF. In conclusion, optimum RLDL gel may be an effective breast cancer treatment.

## 1. Introduction

Breast cancer is a type of cancer that has uncontrolled breast cell proliferation. In women, breast cancer is the most frequent malignancy that leads to death [1]. Early menarche, late menopause and obesity all increase women’s risk factors for breast cancer [2,3]. Although chemotherapeutic drugs have been widely used to treat breast cancer, they are unable to distinguish between cancerous and noncancerous cells, which lead to hazardous side effects [4]. Targeted drug delivery, such as nanoparticles, for the treatment of breast cancer offers a lot of promise, both therapeutically and in pharmacological research. Therapeutic agents are delivered in a targeted and controlled manner by nanoparticles [5]. Targeting neoplastic cells using nanoparticles improves bioavailability, effectiveness, and selectivity [6].

The most often employed nanoparticles are liposomes which have been studied for the topical treatment of cancer [7]. Liposomes are drug delivery systems consisting of phospholipids and cholesterol dispersed in an aqueous medium [7]. Because of their diffusion capabilities and ability to deliver drugs in a targeted and controlled manner, liposomes are ideal drug delivery carriers. Liposomes, on the other hand, are prone to drug leakage and have low stability, skin penetration capabilities and sometimes phospholipids undergo oxidation and hydrolysis-like reactions [8,9]. New generations of deformable liposomal systems have been introduced in an attempt to increase the systemic absorption of drugs via improving dermal permeation of liposomes. Deformable liposomes are composed of phospholipids and penetration enhancers such as propylene glycol (PG) [8,10]. The addition of PG during the preparation of liposomes could increase the amount of drug permeated, resulting in better efficacy [11]. Transdermal drug delivery is an ideal route for breast cancer treatment compared with the oral route due to the potential reduction in adverse effects, the increased local concentration of the drug and avoidance of first-pass metabolism [12,13].

Raloxifene (RXF), an oral oestrogen receptor antagonist, has been employed as a model drug. RXF is used to prevent and treat invasive breast cancer in postmenopausal women [14,15]. This study aimed to improve RXF antitumor efficacy, bioavailability, and targeting as a potential breast cancer treatment by developing a stable deformable liposome formulation, as well as to investigate the change of liposomal physic-chemical characteristics and penetration upon addition of PG. Different deformable liposome formulations were prepared and characterised in vitro to investigate the deformable liposomal physic-chemical characteristics and penetration. In order to investigate bioavailability, antitumor effectiveness and permeation, the optimum formulation was selected and included in a carbopol gel.

## 2. Materials and Methods

### 2.1. Materials

Agitech Company in Egypt provided cholesterol, phospholipid (phospholipon 90 G) and chloroform. Other components were purchased from Cornell Lab in Egypt, including propylene glycol (PG) and carbopol 974.

### 2.2. Box–Behnken Design

The Box–Behnken design was applied to prepare different Raloxifene-loaded deformable formulations (RLDL) (Table 1) using Design-Expert software (version 13, Stat-Ease Inc., Minneapolis, MA, USA). The independent variables were concentrations of propylene glycol (0–10% *w/w*, X_1_), phospholipid (1–3% *w/w*, X_2_) and cholesterol (0.05–0.15% *w/w*, X_3_). Entrapment efficiency (EE, Y_1_), particle size (nm, Y_2_), zeta potential (mV, Y_3_) and the steady-state flux (Fss, µg/cm^2^/h, Y_4_) were selected as dependent variables.

### 2.3. Preparation of Raloxifene-Loaded Deformable Liposome Formulations

A method of thin hydration was used to prepare different RLDL formulations [16]. Phospholipid, PG, RXF (10 mg) and cholesterol were dissolved in chloroform and rotary evaporated (RE300, Mamhilad, UK) at 40 °C under a vacuum pump. The formed lipid film was then hydrated for 1 h at 40 °C in 10 ml of isotonic phosphate buffer (IPB). The prepared RLDL was sonicated (Sonix TV, SC) and then cooling centrifuged (SIGMA 3-30 K, Steinheim, Germany) for 1 h at 15,000 rpm to separate RLDL pellets from unentrapped RXF. The RLDL pellets were washed with IPB and recentrifuged to separate all unentrapped RXF. The collected RLDL pellets were resuspended again in 10 ml of IPB and stored at 4 °C.

### 2.4. In Vitro Characterization of RLDL Formulations

#### 2.4.1. Entrapment Efficiency Determination

An indirect method was used to determine entrapment efficiency of RLDL formulations. The RLDL suspension was centrifuged (15,000 rpm, 1 h, 4 °C) and then the entrapped RXF was dissolved in methanol. The entrapment efficiency was determined using a spectrophotometer (JascoV530, MD) at 293 nm as follows [17]:

% EE = Entrapped RXF content/initial RXF content × 100(1)

#### 2.4.2. Zeta Potential and Particle Size Determination

Dynamic light scattering (Malvern, Germany) was used to measure the size, poly-dispersity index and zeta potential of different Raloxifene-loaded deformable liposome formulations in three replicates. The experimental parameters for dynamic light scattering are water as a dispersant with a refractive index of 1.330, a dielectric constant of 78.5, a temperature of 25 °C and a viscosity of 0.8872 cP. The refractive index of the materials was 1.00 with an absorption of 0.200 and a detection angle of 15 °C [18].

#### 2.4.3. Ex Vivo Drug Permeation and Skin Deposition Studies

The permeation of different RLDL formulations and RXF suspensions was investigated using a donor compartment of Guinea Pig skin diffusion cell (5 cm^2^) [19]. To comply with RXF’s sink condition, a dissolution medium containing 50 mL of IPB + 0.1% *w*/*w* of Tween 80 was used. At 100 rpm and 37 ± 0.5 °C, the dissolution equipment (Hanson, MA, USA) was set. The donor compartment received a volume of deformable liposomal suspension equivalent to 1 mg of RXF, which was subsequently immersed in the dissolution medium. At predefined time points, samples of different RLDL formulations were taken and replaced with an equal volume. A spectrophotometer (JascoV530, MD) was used to analyse the permeation samples at 293 nm. In triplicate, the transdermal flux (Fss) was computed as follows [19]:

Fss = (The permeation rate)/(The active diffusion area)(2)

At the end of the permeation investigation, the skin was sliced (slices of 5 µ) and combined with IPB + 0.1% *w*/*w* of Tween 80 to assess the skin deposition of RXF from different RLDL formulations and RXF suspension [20]. To achieve total RXF release, the skin pieces were homogenised at 8000 rpm for 10 min using a high-shear homogenizer (DI 25 basic, IKA, Staufen, Germany). The skin homogenyate was centrifuged at 10,000 rpm for 5 min before being analysed in triplicate using a spectrophotometer at 293 nm.

### 2.5. Optimization of RLDL Formulations

Design expert software was used to analyse all quantative of Y_1_–Y_4_ using the ANOVA test with *p*-values less than 0.05. It provides a model matrix for evaluating the best fitted model and polynomial equations (Equations (5)–(8)) for evaluating the mathematical relationships between independent variables. Additionally, it provides 3D graphs for assessing the impact of independent variables on Y_1_–Y_4_. After analysis of all quantative outcomes of Y_1_–Y_4_, the point prediction method was used to select the optimum formulation [21]. In this study, the optimum formulation was chosen based on the criterion of maximising % EE and Fss while maintaining a small vesicle size.

### 2.6. In Vitro Characterization of the Optimum RLDL Formulation

#### 2.6.1. Thermal Analysis Studies

Differential scanning calorimetry (DSC-60F3, NETZSCH-Geratebau GmbH, Maia, Germany) was used to examine the thermal analysis of the RXF, phospholipid, cholesterol, and RLDL [22]. Samples (3–5 mg) were accurately weighed into 50 µL DSC aluminium pans with a thickness of 0.1 mm. DSC thermograms were done at a heating rate of 5 °C/min and a nitrogen gas flow rate of 25 mL/min. After heating the samples to 300 °C, they were quickly cooled to 25 °C.

#### 2.6.2. STEM Measurements

The scanning transmission electron microscope (STEM, Carl Zeiss, Jena, Germany) was used to evaluate the RLDL’s appearance [23]. 20 µL of vesicle suspension were poured over a carbon-coated copper grid (300 mesh size) and allowed to dry to allow the formulation to adhere to the carbon substrate. The film was examined at appropriate magnifications under STEM (70 kV voltage).

#### 2.6.3. In Vitro Drug Release Studies

The release of the optimum RLDL formulation was determined using the Hanson dissolution apparatus. The dialysis bag was filled with a volume of deformable liposomal suspension equivalent to 1 mg of RXF and mixed in 50 mL of IPB (pH 7.4) + 0.1% *w*/*w* of Tween 80. At 100 rpm and 32 ± 0.5 °C, the dissolution apparatus (Hanson, MA, USA) was set. At predefined time points, samples of RLDL formulation were taken and replaced with an equal volume. A spectrophotometer (JascoV530, MD) was used to analyse the release samples at 293 nm as follows [21]:

%Release = Released RXF content/initial RXF content × 100(3)

#### 2.6.4. Drug Release Kinetics

Using the DDSolver computer software, the kinetic of RLDL’s release was investigated [24,25]. The DDSolver provides a model matrix of forty models for evaluating the best fitted model using the coefficient of determination (R2), Akaike information criterion (AIC), and model selection criterion (MSC) criteria. The model that best fits RXF’s release mechanism has the minimum AIC and the highest R2 and MSC. Similarly, the mechanism of RLDL’s release was investigated by calculating the “n” of the Korsmeyer–Peppas equation [26]. Fickian diffusion was RLDL’s release mechanism. If n = 0.5, a non-Fickian diffusion was RLDL’s release mechanism if 0.5 < n < 1. Furthermore, the DDSolver programme depicts the similarity between the RXF release profile from RLDL formulation and free RXF by calculating the similarity factor "f_2_". The difference in dissolution profiles was insignificant (*p* < 0.05) if f_2_ > 50; the difference in dissolution profiles was significant (*p* < 0.05) if f_2_ < 50. 

#### 2.6.5. Stability Studies

The change in particle size and EE of the optimum RLDL formulation were investigated to determine its stability [27]. The optimum formulation was stored for three months at three different temperatures (4 °C, 25 °C, and 40 °C), with a sample analysed for size and EE in triplicates per month.

### 2.7. Preparation and In Vitro Characterization of Optimum RLDL Formulation Gel

#### 2.7.1. Preparation of Optimum RLDL Formulation Gel

The gel base was prepared by vigorously stirring 2% *w*/*w* of carbopol 974 into water [11]. Triethnolamine was employed to alter the pH of the gel base. The control gel was prepared by vigorously stirring free RXF into the carbopol gel base, whereas the optimum RLDL gel formulation was prepared by vigorously stirring the optimum RLDL formulation into the carbopol gel base. The prepared gel formulations were refrigerated at 4°C after manufacture.

#### 2.7.2. In Vitro Characterization of Optimum RLDL Formulation Gel

A Brookfield viscometer (DV-III, USA) was used to determine the viscosity coefficient of the produced gel formulations [22]. The viscosity coefficient was estimated using the following formula from the log shear rate vs log shear stress.

Log (shear stress) = N log (shear rate) − log (viscosity coefficient)(4)

In vitro, the permeation of optimum RLDL gel and control gel was determined as described previously.

### 2.8. In Vivo Antitumour Characterization of the Optimum RLDL Gel

#### 2.8.1. Study Design

At 22 ± 2 °C and 50 ± 5% humidity, a complete diet and water were provided to 48 mature female mice (200–300 g). Before being utilised in experiments, the mice were given a week of acclimatisation and were given typical conditions such as free access to water, a healthy meal, and clean cages. The dorsal skin of each animal was clipped 48 hours before the experiment to remove a 3 × 3 cm^2^ section. To create a tumour, each mouse was given a single dose of the tumour initiator DMBA (1 mg in 200 L acetone) [28]. Following the injection of DMBA, epidermal tumours became bigger and more commonly proceeded to malignant carcinoma (known as papilloma). This procedure was approved by the animal ethical committee of Beni-Suef University’s Faculty of Pharmacy.

#### 2.8.2. Animals

Mice were divided into five groups, each with six animals, at random. G1 was assigned to the positive control group. Oral RXF, control gel, RXF-loaded liposome gel containing 0% PG and optimum RLDL gel formulation were given to G2, G3, G4 and G5 groups, respectively. This procedure was approved by the animal ethical committee of Beni-Suef University’s Faculty of Pharmacy.

#### 2.8.3. Antitumour Activity and Toxicity Determination

The quantification of the number and diameter of papilloma > 1 mm were investigated weekly to measure the antitumour activity of the RLDL formulation [28]. Additionally, histopathology estimation was obtained to confirm the antitumour activity and toxicity of the RLDL formulation. All mice in each group were anaesthetized and slaughtered at the end of the experiment. The tumour samples were fixed in buffered formalin before being sliced and stained with haematoxylin-eosin for histological evaluation [29].

#### 2.8.4. In Vivo Permeation and Bioavailability Studies

Compared to RXF-loaded liposome gel containing 0% PG, the in vivo skin penetration of RLDL gel formulation was measured to confirm the enhancement effect of PG on the liposome’s permeation. Eighteen adult female mice weighing 200–300 g were divided into three groups, each with six mice. Oral RXF, RXF-loaded liposome gel containing 0% PG and optimum RLDL gel formulation were given to G1, G2, and G3 groups, respectively. Blood samples were collected in EDTA tubes at various time intervals for 24 hours after administration and centrifuged at 3.0× *g* for 10 min, after which plasma was separated and analysed using the HPLC method [30]. An isocratic separation of RXF was obtained through analytical column C-18 with dimensions of 150 × 4.6 mm using a 67:33 v/v mobile phase made up of a buffer solution (pH 3) of orthophosphoric acid and acetonitrile. The detection of RXF was carried out at 287 nm, with a mobile phase flow rate of 1 mL/min and a 10 µL injection volume. The linearity was obtained between 0.125 and 5 μg/mL (R^2^ = 0.999) with a retention time of 4.75 min. Samples of plasma were combined with acetonitrile before being centrifuged for 10 min at 3.0× *g*. The supernatant was evaporated and dissolved in the mobile phase before being analysed in triplicate by HPLC to determine the total amount of RXF penetrated [31]. Non-compartmental analysis was carried out using the WinNonlin software (version 1.5, Jersey, NJ, USA) [18]. The area under the concentration time curve was calculated using the linear trapezoidal method (AUC). The maximum concentration (Cmax) and maximum time (Tmax), mean residence time (MRT), elimination rate constant (K) and biological half-life (t_1/2_) were determined using the plasma concentration vs. time profile. The student’s t-test was used to assess the statistical analysis.

Additionally, the in vivo skin deposition of RLDL gel formulation was measured compared to RXF-loaded liposome gel containing 0% PG. The skin of the second and third groups was collected and the stratum corneum was removed using the tape stripping technique. The skin was the homogenised for 10 min at 8000 rpm and the tissue homogenate (1 mL) was mixed with acetonitrile before being centrifuged for 10 min at 3.0× *g* [32]. The supernatant was evaporated and dissolved in the mobile phase for HPLC analysis in triplicate to determine the drug concentration [28]. The student’s t-test was used to assess the statistical analysis.

### 2.9. Statistical Analysis

The data was statistically analysed using De-sign-Expert software and IBM-SPSS Statistics (version 22, Armonk, NY, USA) using the ANOVA test (*p* < 0.05). A mean and standard deviation (SD) were used to present the data.

## 3. Results and Discussion

### 3.1. Preparation of Raloxifene-Loaded Deformable Liposoms Formulations

Liposomes, on the other hand, are a promising approach to improving anticancer topical delivery, but confocal microscopy revealed that they had minimal skin penetration characteristics [8,9]. Addition of PG to the initial liposome formulation to produce deformable liposomes improves liposome permeability and retention, which is important for anticancer administration [8,10]. The thin film hydration process was used to make all of the RLDL formulations. For small-scale deformable liposome production in a research laboratory, one of the simplest methods is thin film hydration [9,11]. Based on data collected from literature reviews, altering the phospholipid, cholesterol and PG concentrations are critical for deformable liposomes preparation [8,10,33]. Preliminary experiments were carried out, according to literature reviews, to determine the independent variable concentration ranges required for this study [8,10,33]. Phospholipids contribute to the formation of lipid bilayers [9,34,35]. According to preliminary experiments, increasing the phospholipid concentration resulted in increasing the % EE and particle size. However, the relationship holds true only up to a phospholipid concentration of 3%, after which further increases result in larger vesicles with no effect on entrapment efficiency. These findings were agreed with Abdulbaqi, I.M., et al. [11]. Cholesterol is a stiff molecule that improves the stability and rigidity of the lipid bilayer [8,33,36,37]. According to preliminary trials, increasing the cholesterol concentration produced stable deformable liposomal vesicles with a high % EE. However, the relationship is true only until a concentration of 0.3%, because cholesterol competes with drug producing vesicles with low EE. These findings were agreed with Abdulbaqi, et al. [11]. PG increases vesicle flexibility and stability by acting as a penetration enhancer and –ve charge provider [12]. It’s also employed as an edge activator in liposome production to create a more fluid bilayer with a small particle size [8,11]. However, the relationship holds true only up to a PG concentration of 10%, after which further increases result in leakage of the encapsulated drugs out of vesicles. These findings were agreed upon by Gomez et al. [33]. Successfully, different RLDL formulations containing concentrations of propylene glycol (0–10% *w*/*w*, X_1_), phospholipid (1–3% *w*/*w*, X_2_) and cholesterol (0.05–0.15% *w*/*w*, X_3_) as constructed in Table 1 were prepared.

Design expert software was used to analyse all quantative outcomes of Y_1_–Y_4_. The quadratic model was the best fitted model for all dependent variables with *p* < 0.05. Mathematical relationships between independent variables were evaluated using the following polynomial equations:

% EE = +64.28 + 8.04X_1_ + 14.74X_2_ + 5.00X_3_ + 0.78X_1_X_2_ + 0.77X_1_X_3_ + 1.18X_2_X_3_ − 0.25X_1_^2^ − 0.39X_2_^2^ − 0.61X_3_^2^ (5)

Vesicle size = +257.97 − 47.00X_1_ + 83.97X_2_ − 24.83X_3_ + 3.06X_1_X_2_ − 3.67X_1_X_3_ + 2.98X_2_X_3_ + 3.04X_1_^2^ − 5.45X_2_^2^ − 1.52X_3_^2^ (6)

Zeta Potential = −25.32 − 6.71X_1_ − 11.57X_2_ − 3.55X_3_ − 1.23X_1_X_2_ − 0.37X_1_X_3_ − 0.25X_2_X_3_ + 0.36X_1_^2^ + 0.14X_2_^2^ + 0.73X_3_^2^ (7)

Fss = +3.04 + 0.62X_1_ − 0.94X_2_ − 0.26X_3_ + 0.11X_1_X_2_ + 0.061X_1_X_3_ − 0.059X_2_X_3_ − 0.075X_1_^2^ − 0.18X_2_^2^ + 0.055 X_3_^2^ (8)

### 3.2. In Vitro Characterization of RLDL Formultionse

#### 3.2.1. Entrapment Efficiency Determination

**Figure 1:**
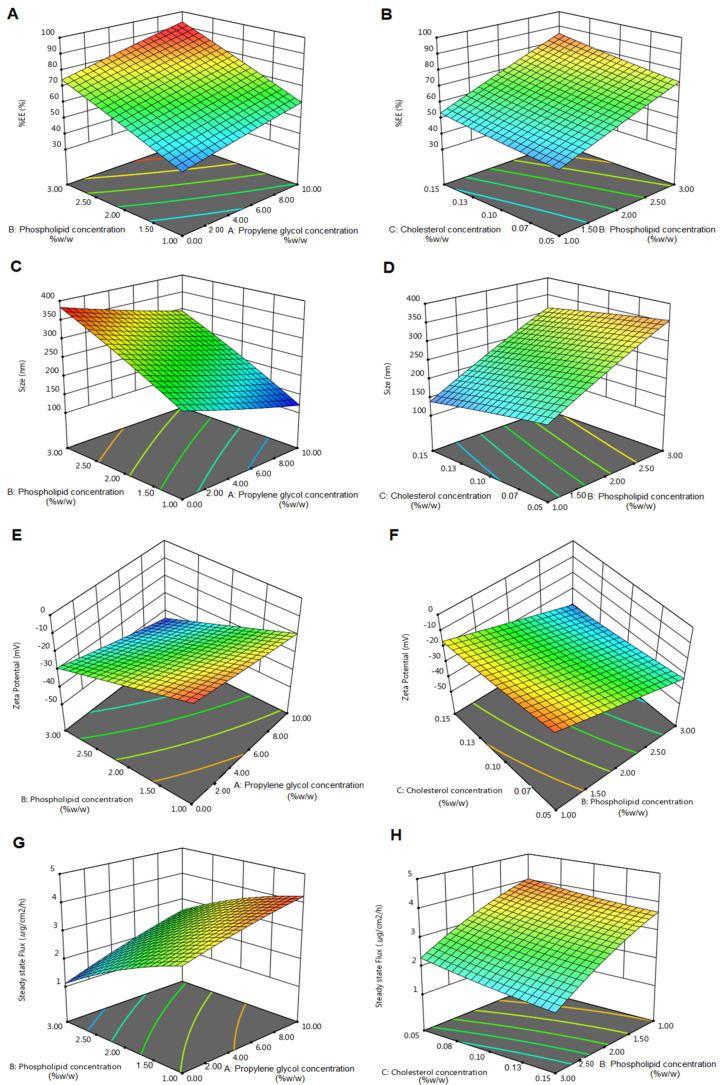
3D response surface plots showing the effect of phospholipid and PG concentrations and the effect of phospholipid and cholesterol concentrations on % EE (**A**,**B**), particle size (**C**,**D**), zeta potential (**E**,**F**) and steady state flux (**G**,**H**).

A spectrophotometric method in the UV/Vis region has been developed to quantify RXF. The RFX calibration curve was spectrophotometrically (JascoV530, Easton, MD, USA) obtained at wavelength of 293 nm. The quantitative method of measuring RXF was reliable because there was a linear relationship between absorbance and RXF concentration that obeys the law of Beer Lambert with a coefficient of determination (R^2^) of 0.999. The drug content of the produced formulations was calculated using the % EE. The % EE was found to range from 41.31 ± 0.95% to 87.53 ± 1.10%. Figure 1A,B shows that % EE was significantly (*p* < 0.05) increased by increasing X_1_ because drug solubility and distribution within the vesicle are improved by PG [8,38]. % EE was significantly (*p* < 0.05) increased by increasing X_2_ because phospholipids produce stable multilamellar vesicles with rigid bilayers [8,10,33]. Additionally, % EE was significantly (*p* < 0.05) increased by increasing X_3_ because cholesterol is a stiff molecule that improves the stability and rigidity of the lipid bilayer [39].

#### 3.2.2. Zeta Potential and Particle Size Determination

The results of dynamic light scattering revealed a uniform distribution of vesicles with a low polydispersity index. Figure 1C,D shows that the size was significantly (*p* < 0.05) decreased by increasing X_1_ and X_3_, where F1 had a vesicle size of 329.80 ± 4.51 nm with a PDI of 0.431 ± 0.03 and F2 had a vesicle size of 242.80 ± 5.07 nm with a PDI of 0.251 ± 0.02. Additionally, the size was significantly (*p* < 0.05) increased by increasing X_2,_ where F3 had a vesicle size of 295.33 ± 2.60 nm with a PDI of 0.263 ± 0.05 and F13 had a vesicle size of 122.13 ± 2.63 nm with a PDI of 0.194 ± 0.01. The zeta potential method is employed in order to determine the surface charge of nanoparticles [40]. The colloidal stability of the produced formulations can be predicted by the zeta potential. The zeta potential was found to range from −44.30 ± 0.20 mV to −7.80 ± 0.36 mV. Figure 1E,F shows that the stability was significantly (*p* < 0.05) increased by decreasing X_1_, X_2_ and X_3_. These outcomes were obtained because of the tendency of phospholipon 90 G to coalesce. Increasing phospholipon 90 G concentration increases the aggregation of the vesicles. Cholesterol is a rigid molecule which is incorporated during deformable liposome formulation to improve the stability and rigidity of the lipid bilayer. Electrostatic repulsion is increased and vesicle aggregation is reduced when the negative charge of the vesicles is increased [11].

#### 3.2.3. Ex Vivo Drug Permeation and Skin Deposition Studies

**Figure 2:**
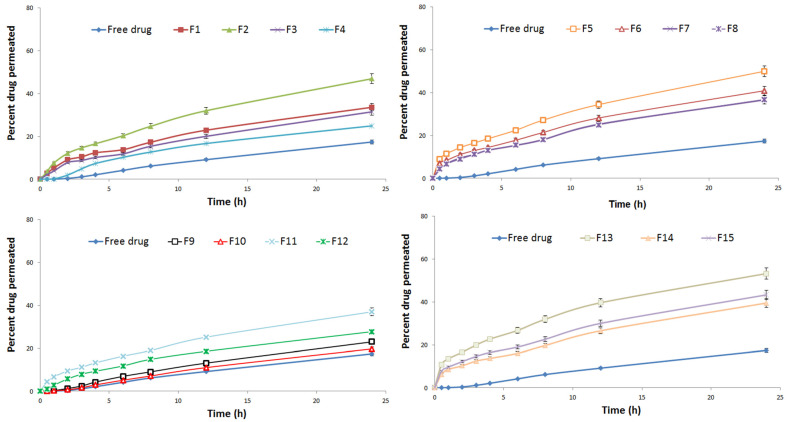
In vitro permeation profiles of RXF from free RXF and different RLDL formulations (*n* = 3 ± SD).

The dissolution volume for RXF release and permeation studies from RLDL formulations was determined by calculating RXF’s saturated solubility. Because RXF’s solubility is 0.18 mg/mL, a dissolution medium containing 50 ml of IPB + 0.1% *w*/*w* of Tween 80 has a high solubility enough to satisfy the sink condition. The release was studied and found to range from 39.24 ± 0.47% to 66.92 ± 0.50%. The enhancement in release was significantly (*p* < 0.05) achieved by increasing X_1_ and decreasing X_2_ and X_3_. Additionally, the permeation profile of different RLDL formulations was studied as shown in Figure 2. When compared to different RLDL formulations, RXF permeation from free RXF was significantly (*p* < 0.05) lower. Figure 1G,H shows that the enhancement in permeation and mucosal flux were significantly (*p* < 0.05) achieved by increasing X_1_ and decreasing X_2_ and X_3_. The skin deposition was studied and found to range from 83.52 ± 0.97 to 155.17± 0.50 µg/cm^2^. The decrease in skin deposition was significantly (*p* < 0.05) achieved by increasing X_1_ and decreasing X_2_ and X_3_. These outcomes were obtained because phospholipids contribute to the formation of lipid bilayers [9]. Cholesterol is a stiff molecule that improves the stability and rigidity of the lipid bilayer [11,41]. They prevent leakage and reduce the permeability of vesicles [42]. Increasing the negative charge of deformable liposomes increases vesicle flexibility and creates a more fluid bilayer [8,11].

### 3.3. Optimization of RLDL Formulations

After analysis of all quantitative outcomes of Y_1_–Y_4_, the point prediction method was used to select the optimum formulation. In this study, the optimum formulation was that including propylene glycol (10% *v*/*v*), phospholipid (1.40% *w*/*w*) and cholesterol (0.15% *w*/*w*) and displayed a % EE of 78.34 ± 1.04%, a particle size of 128.33 ± 3.14 nm, a zeta potential of −27.01 ± 0.66 mV and a steady-state flux of 4.21 ± 0.02 µg/cm^2^/h.

### 3.4. In Vitro Characterization of the Optimum RLDL Formulation

#### 3.4.1. Thermal Analysis Studies

**Figure 3:**
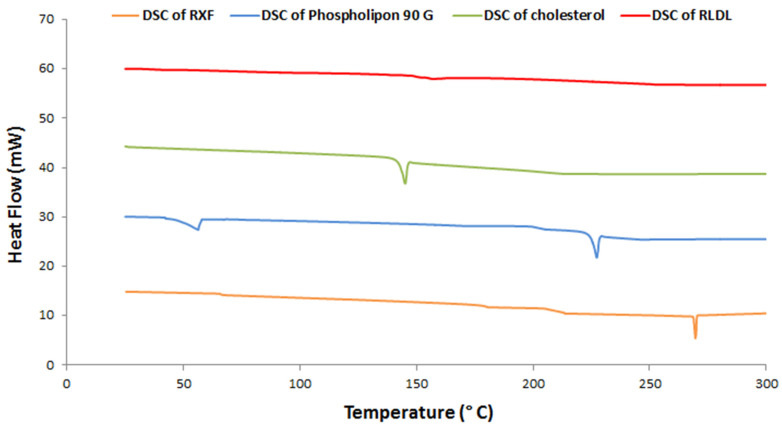
DSC thermogram of optimum RLDL formulation components.

A DSC analysis was obtained to measure the thermal behaviour of different RLDL formulation components as shown in Figure 3. During the crystallisation process, DSC can also identify probable polymorphic transitions. The DSC studies showed an endothermic peak for RXF at 269.9 °C. The phospholipid thermogram shows endothermic peaks at 51 and 224 °C. The cholesterol thermogram shows an endothermic peak at 144.46 °C. When RXF, cholesterol and phospholipid were mixed together, their crystallinity was reduced because they formed a bilayer. RXF’s crystallinity was diminished upon mixing them in the formulation because RXF integrated into the bilayer in an amorphous form.

#### 3.4.2. STEM Measurements

**Figure 4:**
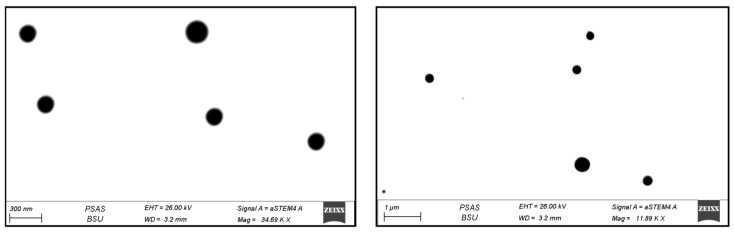
RLDL formulation STEM morphology in various magnifications.

Figure 4 shows several magnifications of the RLDL formulation STEM morphology. The deformable liposomes are spherical nanovesicles that appear as black dots.

#### 3.4.3. Stability Studies

**Figure 5:**
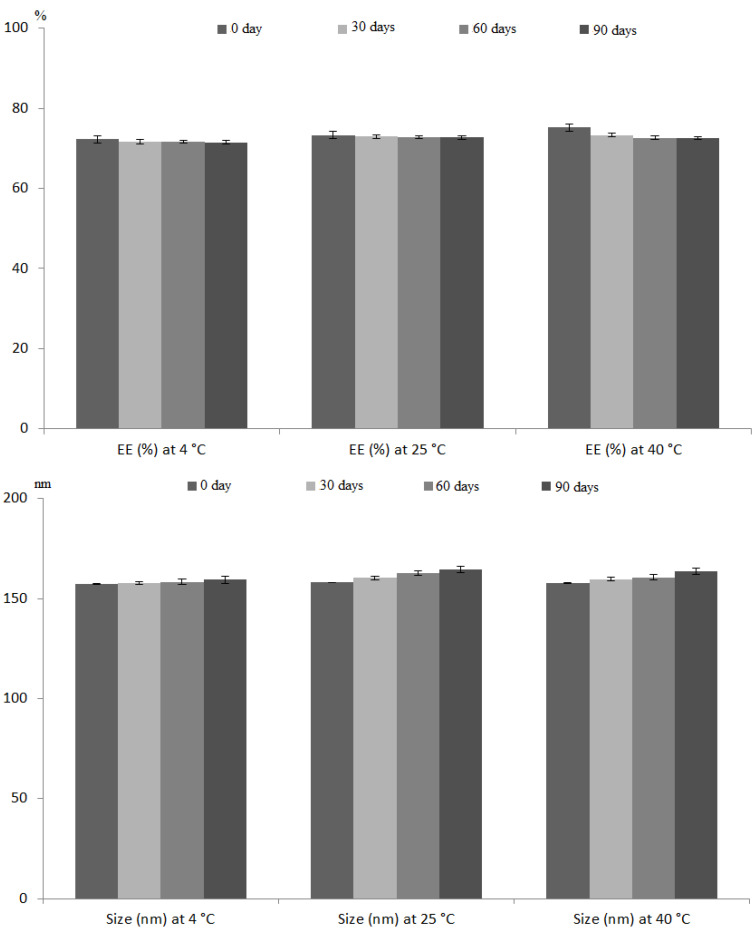
The effect of storage on the % EE and particle size of the optimum RLDL formulation at 4 °C, 25 °C and 40 °C. Each value was the mean ± standard deviation of measurements from three samples.

Figure 5 depicts the evaluation of the RLDL formulation’s stability. The change in % EE and size of RLDL was insignificant (*p* > 0.05) using ANOVA test at 4 °C, 25 °C and 40 °C. 

#### 3.4.4. In Vitro Drug Release Kinetic Studies

When compared to optimum RLDL formulation, RXF release from free RXF was significantly (*p* < 0.05) higher, where the release of RXF from free RXF and RLDL formulation was found to be 98.79 ± 0.52% and 60.71 ± 0.57%, respectively. DDSolver provides a model matrix for evaluating the best fitted model [24,25]. The Korsmeyer–Peppas model was the best fitted model describing the RLDL’s release because it gave the minimum AIC (17.3727), and maximum R^2^ (0.9985) and MSC (5.8935). Because Korsmeyer–Peppas’s “n” is 0.441 ± 0.005, a Fickian diffusion was RLDL’s release mechanism. Table 2 depicts the similarity between the RXF release profile from RLDL formulation and free RXF. Because the similarity factor (f_2_) is 34.12 ± 2.65, the difference in dissolution profiles was significant (*p* < 0.05). These outcomes confirmed that the deformable liposomes delayed RXF release in a controlled manner.

### 3.5. Preparation and In Vitro Characterization of RLDL Gel Formulations

Carbopol is a buffering anionic polymer that helps to maintain the desired pH while causing no skin irritation [43,44,45]. Carbopol polymer provides the required viscosity and bio-adhesive properties when combined with deformable liposomes [11,41]. A carbopol gel was successfully combined with the optimal RLDL. The viscosity coefficient of the RLDL gel and control gel was 147.82 ± 0.73 and 156.12 ± 0.64 cP, respectively. The inclusion of PG could account for the RLDL gel’s slight decrease in viscosity coefficient. Additionally, RLDL’s release and permeation were significantly decreased upon incorporation of RLDL into the carbopol gel. The release and permeation were retarded up to 52.54 ± 0.79% and 102.88 ± 1.01 µg/cm^2^, respectively within 24 h with a mucosal flux of 3.91 ± 0.01 µg/cm^2^/h and skin deposition of 98.71 ± 0.78 µg/cm^2^. As a result of Carbopol gel’s cross-linking; the skin permeation of deformable liposomes was decreased [44].

### 3.6. In Vivo Antitumour Characterization of the Optimum RLDL Gel

#### 3.6.1. Antitumour Activity and Toxicity Determination

**Figure 6:**
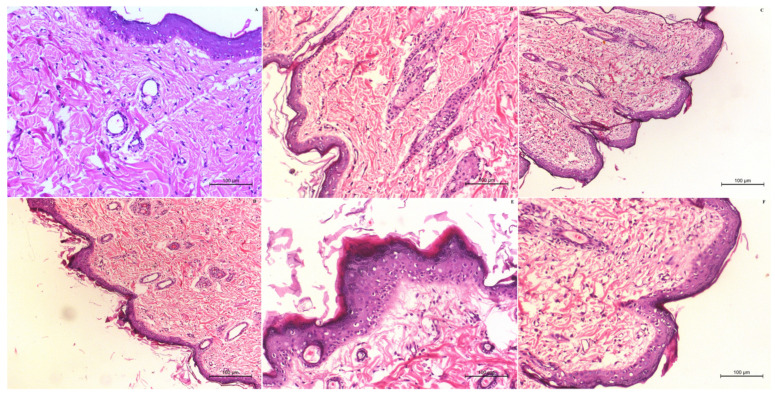
Histological examination of the –ve control group (**A**), +ve control group (**B**), control gel group (**C**), oral RXF suspension (**D**), RXF-loaded liposome gel containing 0% PG (**E**) and optimum RLDL gel (**F**).

The antitumor activity was investigated by the quantification of the number and diameter of papilloma > 1 mm weekly. When compared to oral RXF suspension and RXF-loaded liposome gel containing 0% PG, the optimum RLDL formulation resulted in a significant (*p* < 0.05) decrease in the number and diameter of papilloma. The presence of malignant proliferative epithelial cells was discovered histopathologically in the +ve control group. This group had some vacuolated epithelial cells. Dermal granulation tissue appeared as a result of vascular proliferation. Additionally, hyperkeratosis, inflammatory cell infiltrations and signs of dermal toxicity appeared with oedema. The free RXF gel group showed no improvement in symptoms of skin toxicity in the dermis and epidermal layers (Figure 6C). Even following treatment with oral RXF, the size and number of papilloma increased but at a slower rate than the +ve control group (Figure 6D). In the dermal subcutaneous layer, the epidermis’ surface epithelium displayed hyperkeratosis and acanthosis, as well as a disseminated inflammatory reaction and edoema. The existence of a chronic proliferative reaction is indicated by hyalinosis of certain blood vessels (Angiopathic). RXF-loaded liposome gel with 0% PG gel group (Figure 6E) showed a moderate improvement in skin toxicity in terms of papilloma size and number. Despite the fact that the number and infiltration rates of inflammatory cells have improved in Figure 6E, hyperplasia in the skin persists. When compared to the RXF-loaded liposome gel containing 0% PG gel group, the optimum RLDL gel group Figure 6F exhibited a significant reduction in hyperplasia and hyperkeratosis. When compared to the –ve control group (mice received neither DMBA nor SNG, Figure 6A), the optimal RLDL gel group demonstrated an absence of inflammatory reactions inside the dermis, as well as a significant improvement in skin toxicity, with normal architecture, structure, and appearance in both the epidermal and dermal layers. Optimum RLDL gel showed improved anti-angiogenic and antitumor effectiveness when compared to oral RXF at the same dose. The papilloma were elucidated when RXF was introduced into deformable liposome gel carriers due to the targeting capabilities and sustained action of deformable liposome gel carriers. 

#### 3.6.2. In Vivo Permeation and Bioavailability Studies

**Figure 7:**
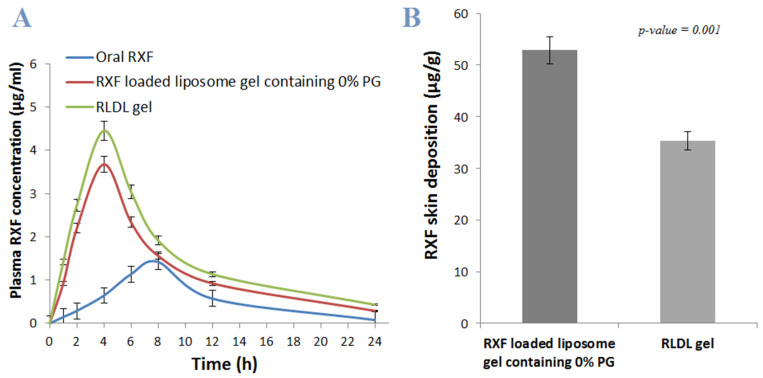
(**A**) Plasma RXF concentration (μg/mL) after oral and transdermal administration; (**B**) RXF concentration in the skin after application of RLDL gel and RXF-loaded liposome gel containing 0% PG.

The pharmacokinetic parameters were calculated using the plasma concentration-time curve (Figure 7A). Compared to the AUC of oral RXF (30.43 ± 2.53 μg·h/mL), RLDL gel exhibited a significant (*p* < 0.001) high AUC (84.38 ± 3.78 μg·h/mL) with greater relative bioavailability by 2.77 folds. Compared to the AUC of RXF-loaded liposome gel containing 0% PG (51.14 ± 2.52 μg·h/mL), RLDL gel exhibited a significant (*p* < 0.001) high AUC with greater relative bioavailability by 1.65 folds. These outcomes confirmed the enhancement effect of PG on the liposome’s permeation. Avoiding first-pass hepatic metabolism was the cause of enhanced bioavailability of RLDL gel and RXF-loaded liposome gel containing 0% PG. Additionally, Figure 7A illustrates a significant (*p* < 0.001) increase in the value of Cmax for RLDL gels (4.45 ± 1.03 μg/mL) compared to that for oral RXF (1.42 ± 0.74 μg/mL). Furthermore, when RLDL gel was compared to oral RXF, the values of MRT and t_0.5_ increased significantly (*p* < 0.001). Compared to RXF-loaded liposome gel containing 0% PG, the in vivo skin deposition of RLDL gel formulation was measured to confirm the enhancement effect of PG on the liposome’s permeation. Figure 7B shows that the RXF-loaded liposome gel with 0% PG had a significantly (*p* < 0.05) greater skin deposition concentration than the RLDL gel. The extended release of RLDL gel and RXF-loaded liposome gel containing 0% PG into different skint layers before absorption into systemic circulation may be responsible for slow and prolonged absorption of RXF and longer T max, which is related to release data [31].

## 4. Conclusions

This study demonstrated that altering the phospholipid, cholesterol and PG concentrations are critical for deformable liposomes preparation as well as enhancing the liposome’s permeation. As an optimum formulation, RXF was incorporated into a formulation that including propylene glycol (10% *v*/*v*), phospholipid (1.40% *w*/*w*) and cholesterol (0.15% *w*/*w*) and displayed a % EE of 78.34 ± 1.04%, a particle size of 128.33 ± 3.14 nm, a zeta potential of −27.01 ± 0.66 mV and a steady-state flux of 4.21 ± 0.02 µg/cm^2^/h. In order to investigate bioavailability, antitumor effectiveness and permeation, the optimum formulation was included in a carbopol gel. The optimum gel formulation had 2.77 times higher bioavailability and, as a result, considerable antitumor action as compared to oral RXF. In conclusion, optimum RLDL gel may be an effective breast cancer treatment.

## Figures and Tables

**Table 1:** Composition of various RLDL formulations as well as their responses.

Formulation Code	(X_1_) % *w*/*v*	(X_2_) % *w*/*v*	(X_3_) % *w*/*v*	Y_1_ (%) (Mean ± SD)	Y_2_ (nm) (Mean ± SD)	Y_3_ (mV) (Mean ± SD)	Y_4_ (µg/cm^2^/h) (Mean ± SD)
F1	0	2	0.05	52.32 ± 0.84	329.80 ± 4.51	−14.17 ± 0.31	2.72 ± 0.01
F2	10	2	0.05	66.39 ± 0.83	242.80 ± 5.07	−27.10 ± 0.56	3.87 ± 0.02
F3	10	3	0.1	87.53 ± 1.10	295.33 ± 2.60	−44.30 ± 0.20	2.54 ± 0.03
F4	0	2	0.15	61.35 ± 0.96	283.50 ± 2.89	−20.63 ± 0.38	2.05 ± 0.02
F5	5	1	0.05	46.32 ± 0.84	192.43 ± 2.52	−9.73 ± 0.59	4.00 ± 0.03
F6	10	2	0.15	78.50 ± 0.88	181.83 ± 2.51	−35.03 ± 0.40	3.44 ± 0.03
F7	5	2	0.1	64.19 ± 0.53	258.93 ± 6.62	−25.50 ± 0.36	3.06 ± 0.03
F8	5	2	0.1	64.41 ± 0.56	257.87 ± 5.24	−25.23 ± 0.51	3.04 ± 0.03
F9	5	3	0.15	85.05 ± 0.82	315.53 ± 6.24	−39.67 ± 0.51	1.72 ± 0.05
F10	0	3	0.1	69.41 ± 0.94	382.87 ± 2.48	−28.67 ± 0.4	1.08 ± 0.04
F11	5	2	0.1	64.24 ± 0.6	257.10 ± 7.49	−25.23 ± 0.35	3.02 ± 0.01
F12	5	3	0.05	73.27 ± 0.91	355.27 ± 4.9	−32.17 ± 0.45	2.32 ± 0.03
F13	10	1	0.1	56.31 ± 0.93	122.13 ± 2.63	−18.50 ± 0.40	4.27 ± 0.02
F14	0	1	0.1	41.31 ± 0.95	221.90 ± 3.78	−7.80 ± 0.36	3.27 ± 0.02
F15	5	1	0.15	53.38 ± 0.69	140.77 ± 2.08	−16.23 ± 0.31	3.63 ± 0.04

X_1_: PG concentration; X_2_: Phospholipid concentration; X_3_: cholesterol concentration. Y_1_: % EE; Y_2_: vesicle size; Y_3_: zeta potential; Y_4_: the steady state reflux. SD: standard deviation.

**Table 2:** DDSolver parameters for evaluating the differences between RLDL and free RXF dissolution profiles.

Parameter		Value
Symbol	Name	
f_1_	Difference factor	40.5738 ± 3.21
f_2_	Similarity factor	34.1217 ± 2.65
f_1_cp	Difference factor modified by Costa. P	48.7473 ± 4.02
D	Sum of squared mean differences	4306.495 ± 14.43
D1	Mean distance	16.9071 ± 2.10
D2	Mean squared distance	20.7520 ± 2.14
Res1	Rescigno index 1	0.24452 ± 0.03
Res2	Rescigno index 2	0.2448 ± 0.05
Sd	Difference in similarity	0.2503 ± 0.05
DAUC	Difference of area under the profiles	−636.824 ± 9.46
DABC	Area between the profiles	426.730 ± 5.17

## References

[1] Sun Y.-S., Zhao Z., Yang Z.-N., Xu F., Lu H.-J., Zhu Z.-Y., Shi W., Jiang J., Yao P.-P., Zhu H.-P. (2017). Risk Factors and Preventions of Breast Cancer. Int. J. Biol. Sci..

[2] Harris J.R., Lippman M.E., Veronesi U., Willett W. (1992). Breast cancer. N. Engl. J. Med..

[3] Key T.J., Verkasalo P.K., Banks E. (2001). Epidemiology of breast cancer. Lancet Oncol..

[4] Kashanian S., Rafipour R. (2019). A Review on Targeting Nanoparticles for Breast Cancer. Curr. Pharm. Biotechnol..

[5] Yezhelyev M.V., Gao X., Xing Y., Al-Hajj A., Nie S., O’Regan R.M. (2006). Emerging use of nanoparticles in diagnosis and treatment of breast cancer. Lancet Oncol..

[6] Dianzani C., Zara G.P., Maina G., Pettazzoni P., Pizzimenti S., Rossi F., Gigliotti C.L., Ciamporcero E.S., Daga M., Barrera G. (2014). Drug delivery nanoparticles in skin cancers. BioMed Res. Int..

[7] Mezei M., Gulasekharam V. (1980). Liposomes-a selective drug delivery system for the topical route of administration I. Lotion dosage form. Life Sci..

[8] Elsayed M.M., Abdallah O.Y., Naggar V.F., Khalafallah N.M. (2006). Deformable liposomes and ethosomes: Mechanism of enhanced skin delivery. Int. J. Pharm..

[9] Zellmer S., Pfeil W., Lasch J. (1995). Interaction of phosphatidylcholine liposomes with the human stratum corneum. Biochim. Biophys. Acta (BBA)-Biomembr..

[10] Elmoslemany R.M., Abdallah O.Y., El-Khordagui L.K., Khalafallah N.M. (2012). Propylene Glycol Liposomes as a Topical Delivery System for Miconazole Nitrate: Comparison with Conventional Liposomes. AAPS PharmSciTech.

[11] Abdulbaqi I.M., Darwis Y., Khan N.A.K., Assi R.A., Khan A.A. (2016). Ethosomal nanocarriers: The impact of constituents and formulation techniques on ethosomal properties, in vivo studies, and clinical trials. Int. J. Nanomed..

[12] Calienni M.N., Febres-Molina C., Llovera R.E., Zevallos-Delgado C., Tuttolomondo M.E., Paolino D., Fresta M., Barazorda-Ccahuana H.L., Gómez B., Alonso S.D.V. (2019). Nanoformulation for potential topical delivery of Vismodegib in skin cancer treatment. Int. J. Pharm..

[13] Sabale V., Vora S. (2012). Formulation and evaluation of microemulsion-based hydrogel for topical delivery. Int. J. Pharm. Investig..

[14] Mandal U., Mahmood S., Taher M. (2014). Experimental design and optimization of raloxifene hydrochloride loaded nanotransfersomes for transdermal application. Int. J. Nanomed..

[15] Smita N., Sanidhya S., Bhaskar V. (2016). Formulation and evaluation of Raloxifene hydrochloride tablets with improved dissolution profile. Int. J. Adv. Pharm..

[16] Tefas L.R., Sylvester B., Tomuta I., Sesarman A., Licarete E., Banciu M., Porfire A. (2017). Development of antiproliferative long-circulating liposomes co-encapsulating doxorubicin and curcumin, through the use of a quality-by-design approach. Drug Des. Dev. Ther..

[17] Gamal F.A., Kharshoum R.M., Sayed O.M., El-Ela F.I.A., Salem H.F. (2020). Control of basal cell carcinoma via positively charged ethosomes of Vismodegib: In vitro and in vivo studies. J. Drug Deliv. Sci. Technol..

[18] Gamal A., Saeed H., Sayed O.M., Kharshoum R.M., Salem H.F. (2020). Proniosomal Microcarriers: Impact of Constituents on the Physicochemical Properties of Proniosomes as a New Approach to Enhance Inhalation Efficiency of Dry Powder Inhalers. AAPS PharmSciTech.

[19] Salem H.F., Kharshoum R.M., Abou-Taleb H.A., AbouTaleb H.A., AbouElhassan K.M. (2019). Progesterone-loaded nanosized transethosomes for vaginal permeation enhancement: Formulation, statistical optimization, and clinical evaluation in anovulatory polycystic ovary syndrome. J. Liposome Res..

[20] Shen L.-N., Zhang Y.-T., Wang Q., Xu L., Feng N.-P. (2014). Enhanced in vitro and in vivo skin deposition of apigenin delivered using ethosomes. Int. J. Pharm..

[21] Salem H.F., Kharshoum R.M., El-Ela F.I.A., Amr Gamal F., Abdellatif K.R.A. (2018). Evaluation and optimization of pH-responsive niosomes as a carrier for efficient treatment of breast cancer. Drug Deliv. Transl. Res..

[22] Salem H.F., Kharshoum R.M., El-Ela F.I.A., Amr Gamal F., Abdellatif K.R.A. (2020). Treatment of breast cancer with engineered novel pH-sensitive Triaryl-(Z)-olefin niosomes con-taining hydrogel: An in vitro and in vivo study. J. Liposome Res..

[23] Salem H.F., Kharshoum R.M., Sayed O.M., Hakim L.F.A. (2018). Formulation design and optimization of novel soft glycerosomes for enhanced topical delivery of celecoxib and cupferron by Box–Behnken statistical design. Drug Dev. Ind. Pharm..

[24] Zhang Y., Huo M., Zhou J., Zou A., Li W., Yao C., Xie S. (2010). DDSolver: An Add-In Program for Modeling and Comparison of Drug Dissolution Profiles. AAPS J..

[25] Zuo J., Gao Y., Bou-Chacra N., Löbenberg R. (2014). Evaluation of the DDSolver Software Applications. BioMed Res. Int..

[26] Akash MS H., Rehman K., Li N., Gao J.Q., Sun H., Chen S. (2012). Sustained delivery of IL-1Ra from pluronic F127-based thermosensitive gel prolongs its thera-peutic potentials. Pharm. Res..

[27] Nasr M., Mansour S., Mortada N., Elshamy A.A. (2008). Vesicular aceclofenac systems: A comparative study between liposomes and niosomes. J. Microencapsul..

[28] Dias M.F., de Figueiredo BC P., Teixeira-Neto J., Guerra MC A., Fialho S.L., Cunha A.S. (2018). In vivo evaluation of antitumoral and antiangiogenic effect of imiquimod-loaded polymeric na-noparticles. Biomed. Pharmacother..

[29] Aziz RL A., Abdel-Wahab A., El-Ela FI A., Hassan NE H.Y., El-Nahass E.S., Ibrahim M.A., Khalil A.T.A. (2018). Dose-dependent ameliorative effects of quercetin and l-Carnitine against atrazine-induced re-productive toxicity in adult male Albino rats. Biomed. Pharmacother..

[30] Trontelj J., Vovk T., Bogataj M., Mrhar A. (2005). HPLC analysis of raloxifene hydrochloride and its application to drug quality control studies. Pharmacol. Res..

[31] Ammar H.O., Haider M., Ibrahim M., El Hoffy N.M. (2017). In vitro and in vivo investigation for optimization of niosomal ability for sustainment and bioa-vailability enhancement of diltiazem after nasal administration. Drug Deliv..

[32] Praça F.S.G., Medina W.S.G., Eloy J.O., Petrilli R., Campos P.M., Ascenso A., Bentley M.V.L. (2018). Evaluation of critical parameters for in vitro skin permeation and penetration studies using animal skin models. Eur. J. Pharm. Sci..

[33] Abd E., Gomes J., Sales C.C., Yousef S., Forouz F., Telaprolu K.C., Roberts M.S., Grice J.E., Lopes P.S., Leite-Silva V.R. (2020). Deformable liposomes as enhancer of caffeine penetration through human skin in a Franz diffusion cell test. Int. J. Cosmet. Sci..

[34] Schreier H., Bouwstra J. (1994). Liposomes and niosomes as topical drug carriers: Dermal and transdermal drug delivery. J. Control. Release.

[35] Vemuri S., Rhodes C. (1995). Preparation and characterization of liposomes as therapeutic delivery systems: A review. Pharm. Acta Helv..

[36] Bangham A.D., Hill M.W., Miller N.G.A. (1974). Preparation and Use of Liposomes as Models of Biological Membranes. Methods in Membrane Biology.

[37] Franklin R.K., Marcus S.A., Talaat A.M., KuKanich B.K., Sullivan R., Krugner-Higby L.A., Heath T.D. (2015). A novel loading method for doxycycline liposomes for intracellular drug delivery: Characteri-zation of in vitro and in vivo release kinetics and efficacy in a J774A. 1 cell line model of Mycobacterium smegmatis infection. Drug Metab. Dispos..

[38] Akhtar N., Pathak K. (2012). Cavamax W7 Composite Ethosomal Gel of Clotrimazole for Improved Topical Delivery: Development and Comparison with Ethosomal Gel. AAPS PharmSciTech.

[39] Nasseri B. (2005). Effect of cholesterol and temperature on the elastic properties of niosomal membranes. Int. J. Pharm..

[40] El-Ela F.I.A., Hussein K.H., El-Banna H.A., Gamal A., Rouby S., Menshawy A.M., EL-Shimaa E.N., Anwar S., Zeinhom M.M., Salem H.F. (2020). Treatment of Brucellosis in Guinea Pigs via a Combination of Engineered Novel pH-Responsive Curcumin Niosome Hydrogel and Doxycycline-Loaded Chitosan–Sodium Alginate Nanoparticles: An In Vitro and In Vivo Study. AAPS PharmSciTech.

[41] Gamal A., Sayed O., El-Ela F.I.A., Kharshoum R.M., Salem H.F. (2020). Treatment of Basal Cell Carcinoma Via Binary Ethosomes of Vismodegib: In Vitro and In Vivo Studies. AAPS PharmSciTech.

[42] Shaker S., Gardouh A.R.M., Ghorab M.M. (2017). Factors affecting liposomes particle size prepared by ethanol injection method. Res. Pharm. Sci..

[43] Hoare T.R., Kohane D.S. (2008). Hydrogels in drug delivery: Progress and challenges. Polymer.

[44] Jain S., Patel N., Madan P., Lin S. (2016). Formulation and rheological evaluation of ethosome-loaded carbopol hydrogel for transdermal application. Drug Dev. Ind. Pharm..

[45] Poorahmary Kermany B. (2010). Carbopol Hydrogels for Topical Administration: Treatment of Wounds. Master’s Thesis.

